# Radiometric Calibration of a Dual-Wavelength, Full-Waveform Terrestrial Lidar

**DOI:** 10.3390/s16030313

**Published:** 2016-03-02

**Authors:** Zhan Li, David L. B. Jupp, Alan H. Strahler, Crystal B. Schaaf, Glenn Howe, Kuravi Hewawasam, Ewan S. Douglas, Supriya Chakrabarti, Timothy A. Cook, Ian Paynter, Edward J. Saenz, Michael Schaefer

**Affiliations:** 1 Department of Earth and Environment, Boston University, 675 Commonwealth Avenue, Boston, MA 02215, USA; 2 School for the Environment, University of Massachusetts Boston, 100 Morrissey Blvd., Boston, MA 02125, USA; Crystal.Schaaf@umb.edu (C.B.S.); Ian.Paynter@umb.edu (I.P.); Edward.Saenz@umb.edu (E.J.S.); 3 CSIRO Land & Water, GPO Box 1666, Canberra, ACT 2601, Australia; David.Jupp@csiro.au (D.L.B.J.); michael.schaefer@csiro.au (M.S.); 4 Department of Physics and Applied Physics, University of Massachusetts Lowell, 600 Suffolk Street, Lowell, MA 01854, USA; ghowephysics@gmail.com (G.H.); Kuravi_Hewawasam@student.uml.edu (K.H.); Supriya_Chakrabarti@uml.edu (S.C.); Timothy_Cook@uml.edu (T.A.C.); 5 Department of Astronomy, Boston University, 725 Commonwealth Avenue, Boston, MA 02215, USA; douglase@bu.edu; 6 Precision Agriculture Research Group, School of Science and Technology, University of New England, Armidale, NSW 2351, Australia

**Keywords:** terrestrial lidar, vegetation structure, radiometric calibration, DWEL, dual-wavelength lidar, full-waveform lidar

## Abstract

Radiometric calibration of the Dual-Wavelength Echidna^®^ Lidar (DWEL), a full-waveform terrestrial laser scanner with two simultaneously-pulsing infrared lasers at 1064 nm and 1548 nm, provides accurate dual-wavelength apparent reflectance (*ρ_app_*), a physically-defined value that is related to the radiative and structural characteristics of scanned targets and independent of range and instrument optics and electronics. The errors of *ρ_app_* are 8.1% for 1064 nm and 6.4% for 1548 nm. A sensitivity analysis shows that *ρ_app_* error is dominated by range errors at near ranges, but by lidar intensity errors at far ranges. Our semi-empirical model for radiometric calibration combines a generalized logistic function to explicitly model telescopic effects due to defocusing of return signals at near range with a negative exponential function to model the fall-off of return intensity with range. Accurate values of *ρ_app_* from the radiometric calibration improve the quantification of vegetation structure, facilitate the comparison and coupling of lidar datasets from different instruments, campaigns or wavelengths and advance the utilization of bi- and multi-spectral information added to 3D scans by novel spectral lidars.

## 1. Introduction

Light detection and ranging (lidar) is an active remote sensing technique using an instrument that emits coherent laser light. Targets along the laser transmission path scatter the light, and the lidar instrument records the travel time and intensity of the scattered light received by its detector. A new and important application of lidar is the quantification of forest structure, principally measures of the physical dimensions of trees, the amount and location of leaves and gaps between and within tree canopies, through the 3D information acquired by lidar instruments on different remote sensing platforms (terrestrial, airborne and spaceborne). Studies have shown the capability of lidars to facilitate our understanding and management of forest ecosystems [1,2,3,4] by describing the heterogeneity of forest structure in 3D space and its relation to forest function [5,6].

A lidar instrument measures the range to a target through the product of the speed of light and the one-way travel time of light between the instrument and the target. The travel time can be measured using either pulse ranging or continuous wave ranging techniques [1]. For lidar remote sensing of vegetation, which is the application of concern in this paper, pulse ranging lidar is much more commonly used [1], and thus, we will confine our discussion here to pulsing lidar, unless otherwise noted.

Of the spatial location and intensity (while the term “intensity,” as defined in optical physics, refers to the energy flow rate in W·sr^−1^ from a point source of emission, we will use “intensity” here to refer to a measure, normally in digital counts, of the response of the detector-amplifier-digitizer system to the return of energy from a laser pulse or power of a continuous wave scattered by a target into the aperture of the telescope of the lidar instrument and reaching the detector system), the two primary attributes of scattering events recorded by lidar, location by range and zenith and azimuth angles (or as resolved into Cartesian coordinates) has found wide use in the retrieval of forest structural parameters [7], such as diameter at breast height (DBH) [8], tree and canopy height [9,10,11], timber volume [12,13], Leaf Area Index (LAI) [14,15,16,17,18] and others [19,20].

More complete inference of vegetation structure requires using intensity, the other attribute of the return signal. However, intensity information does not provide straightforward interpretation and has been underutilized. Intensities in digital counts output by lidar instruments neither give actual backscattered energy from targets nor relate directly to target physical properties. Accordingly, they are usually processed to remove electronic effects and normalize the decrease of observed intensity with range. The processed intensities thus provide the relative distribution of target return energy from which forest structural parameters can be inferred directly or through empirical regressions, such as gap fraction/probability [21], canopy height profile [22], basal area and aboveground biomass [23] and others [20]. Normalized intensity has also been used in target classification/recognition [14,24,25,26] and estimation of biochemical properties of vegetation [27,28].

Although these studies have documented the usefulness of lidar intensity values, their simple and empirical normalization is primarily arbitrary and only provides “relative reflectance” or “pseudo-reflectance” values through scaling of lidar intensities to adjust the contrast and overall “brightness” of lidar scans [29]. Empirical normalization limits the intercomparison of instruments, makes merging data from two or more instruments or scanning campaigns difficult and causes trouble in the interpretation of normalized intensity values. We need rigorous radiometric calibration to provide a value that is physically interpretable with regard to the properties of targets, such as surface reflectance.

The need for a consistent definition of calibrated lidar intensity is also driven by the recent design or fabrication of bi- or multi-spectral lidar instruments using lasers at different wavelengths or white lasers to exploit the spectral signatures of targets [30,31,32,33,34,35,36,37]. For example, the Dual-Wavelength Echidna Lidar (DWEL) instrument, which is the focus of this paper, uses two coaxial lasers at 1064 nm and 1548 nm wavelengths to differentiate leaves from branches, trunks and ground by taking advantage of the distinctive spectral response of leaves at the two wavelengths [30,38].

For airborne lidar scanning (ALS), recent studies have reviewed the physical concepts of return intensity and radiometric calibration [39,40,41]. Various calibration targets and procedures for ALS data have been proposed and evaluated for several calibration scenarios, including different scanning campaigns with the same instrument [42,43,44], different instruments at the same wavelength [45] or different wavelengths [46].

In terrestrial lidar scanning (TLS), calibrated intensities have been used to measure canopy structure. Examples include retrieval of the multiangle gap probability and then LAI [17], the clumping index estimate [18] and target classification with calibrated intensity alone [47] or along with pulse width from full-waveform data [48]. However, with the exception of a few recent studies on both pulse-ranging TLS [49,50,51] and continuous-wave ranging TLS [52], radiometric calibrations of TLS data are currently scattered among various application studies and are poorly documented or rely on undocumented proprietary calibration algorithms from instrument manufacturers. The radiometric calibration of TLS data faces unique challenges, including: (1) a very large variation in intensity with range that can induce saturation of the detector system by bright targets in the near field and reduced intensities that merge with the noise in the far range; and (2) strong telescopic effects, with defocusing that produces weak signals at near range.

This paper addresses these and other challenges for a dual-wavelength, full-waveform terrestrial laser scanner, the DWEL. No evaluation of the radiometric calibration of TLS data similar to the DWEL has yet been documented for different wavelengths. We present a simultaneous calibration of returns from DWEL’s two lasers, which demonstrates how calibration can ensure both radiometric and spectral fidelity in a unified process, thus providing a pathway for the calibration of other dual- and multi-wavelength terrestrial lidars that may now be in various stages of development and application.

In the paper, we begin with the theoretical lidar equation for canopy structure study using TLS data and then describe the processing of the DWEL waveform data. Our calibration model, based on a generalized logistic function for telescope efficiency and an inverse power fall-off with range, is fitted to stationary scans of panels with different reflectance values at different ranges. We conclude by evaluating the calibration accuracy of dual-wavelength point clouds from DWEL, as well as the sensitivity of the calibration accuracy to errors in both range and intensity measurements.

## 2. Physical Background

### 2.1. Basic Lidar Equation for Forest Canopies

The goal of our radiometric calibration is to obtain range-independent, instrument-independent and physically well-defined measurements for canopy structure modeling and estimation from returned power as detected and recorded by the lidar instrument’s optical and electronic systems. Previous studies [53,54] formulated lidar equations as a function of canopy structure parameters to model large-footprint airborne lidar waveforms, but did not identify a realizable quantity for lidar radiometric calibration. To establish the basis of the lidar calibration for the canopy structure study, we formulate the lidar equation from the basic scattering lidar equation [55] using Ross’s framework of radiation regime modeling of the vegetation canopy [56]. This lidar equation describes the interaction of the laser beam with vegetative elements and identifies the objective variable of the calibration (“apparent reflectance”).

Consider an angular voxel, an elemental volume enclosed by a laser beam between range r and r+Δr from the lidar instrument. Vegetative elements inside one such angular voxel are modeled as a turbid medium composed of tiny thin facets of different orientations in space. We shall not specify the size and thickness of these facets nor their location inside the angular voxel [56]. Laser radiation incident to this angular voxel can be absorbed, reflected back toward the lidar instrument or transmitted through it without interaction with vegetative facets. Simulations of lidar waveforms with Monte Carlo ray tracing have shown that multiple scattering by vegetative elements in the canopy largely has no effect on return waveform shapes and contributes little to return energy, especially for the small laser beam divergence that we use here [57]. Thus, it is reasonable to assume only single scattering in the interaction between laser beams and vegetative facets.

The probability that a laser beam in a given direction reaches an angular voxel at range r without interaction with vegetative facets is given by the gap probability $P_{gap}\left(r\right):$

(1) $$P_{gap}\left(r\right)=e^{-G\int_{0}^{r}u_{L}\left(r^{\prime }\right)dr^{\prime }}$$

where G is the Ross *G*-function, which describes the projection of a unit vegetative area in a given direction; $u_{L}\left(r\right)$ ($m^{2}\cdot m^{-3}$) is the total upper side surface area of all tiny facets (no mutual-shading between facets, *i.e.*, no clumping is considered) within a unit volume at range r along the laser beam [56]. Note that in the expressions below, we will consider only a single laser beam and omit the laser beam direction in $P_{gap}\left(r\right)$ and G.

Let $J_{0}$ be the total outgoing laser radiation energy (units: J) within an infinitesimal time, *i.e.*, an impulse laser energy. The return energy ΔJ (units: J) from the angular voxel between range r to r+Δr received by the telescope of the lidar instrument is:

(2) $$\Delta J=J_{0}P_{gap}\left(r\right)\cdot \Delta \beta \cdot K\left(r\right)\cdot \eta_{sys}\eta_{atm}\;\Delta \beta =\int_{\Omega_{T}=A_{T}/r^{2}}\frac{1}{\pi }\Gamma \left(r,\Omega_{i}\to \Omega_{v}\right)d\Omega_{v}\cdot \int_{r}^{r+\Delta r}u_{L}\left(r^{\prime }\right)dr^{\prime }$$

In these equations, $J_{0}P_{gap}\left(r\right)$ (J) is the laser radiant energy that reaches the angular voxel at range r; Δβ (dimensionless) is the effective backscatter ratio of the angular voxel, *i.e.*, the proportion of the incident laser radiation energy that is scattered back from the angular voxel into the solid angle subtended by the telescope receiving area; the expression $\;\left(1/\pi \right)\Gamma \left(r,\Omega_{i}\to \Omega_{v}\right)$ (${sr}^{-1}$) is the area scattering phase function at range r, *i.e.*, the portion of the radiant energy onto a unit area of vegetative facets in direction $\Omega_{i}$ that is scattered in the direction $\Omega_{v}$ within a unit solid angle, a term that contains both the radiative and structural characteristics of the vegetative facets [56]; $\Omega_{T}$ (sr) is the solid angle subtended by the area of the telescope aperture ($A_{T}$) of the lidar instrument from range r; K(r) (dimensionless) is telescope efficiency at range r (see Section 2.1.3); and $\eta_{sys}$ and $\eta_{atm}$ (dimensionless) are transmission factors that account for energy loss due to the sensor system and atmosphere, respectively.

#### 2.1.1. Apparent Reflectance

In the simplest case: (1) only one angular voxel along a laser beam is filled with vegetative elements at range r, that is $P_{gap}\left(r\right)=1$, and all laser radiation energy of this beam falls onto this voxel; (2) all of the vegetative facets in the angular voxel are Lambertian with the same diffuse reflectance $\rho_{d}$ (dimensionless), and all faces are orthogonal to the laser beam, that is $\left(1/\pi \right)\Gamma \left(\Omega_{i}\to \Omega_{v}\right)=\rho_{d}/\pi$; and (3) all of the vegetative facets together fill the whole laser beam, that is $\int_{r}^{r+\Delta r}u_{L}\left(r^{\prime }\right)dr^{\prime }=1.$ The return energy from such a voxel is then:

(3) $$\Delta J=J_{0}\cdot \rho_{d}\cdot \frac{A_{T}}{\pi r^{2}}\cdot K\left(r\right)\cdot \eta_{sys}\eta_{atm}$$

This simplest case is equivalent to an extended Lambertian panel (by “extended panel,” we describe a panel that is large enough to intercept the whole cross-section of a laser beam at a given range) with reflectance $\rho_{d}$ that fills the laser beam orthogonally at range r. The expression $A_{T}/\left(\pi r^{2}\right)$ in Equation (3) is the proportion of the total hemispherical backscattering that is intercepted by the telescope aperture given this simplest case. Assuming $\eta_{sys}$ and $\eta_{atm}$ are constant with the range of our instrument operation and the laser wavelength in consideration, we may simplify Equation (3) by combining all constants into $\Phi_{0}$ ($J\cdot m^{2}$), *i.e.*,

(4) $$\Delta J=\Phi_{0}\cdot \frac{\rho_{d}\cdot K\left(r\right)}{r^{2}}\;\Phi_{0}=J_{0}\frac{A_{T}}{\pi }\eta_{sys}\eta_{atm}$$

The reflectance $\rho_{d}$, describing the physical properties of targets in the simplest case, is computable from the received energy and also is range independent and instrument independent. For the general case observed in reality, we cannot retrieve the anisotropic reflectance value of each vegetation facet, but may identify a variable called *apparent reflectance*, $\rho_{app}$ (dimensionless), to represent the overall structural and radiative characteristics of all of the vegetative facets as a whole illuminated by a laser beam. The $\rho_{app}$ for the general case is calculated in the same way as $\rho_{d}$ for the simplest case, *i.e.*,

(5) $$\Delta J=\Phi_{0}\cdot \frac{\rho_{app\;}\cdot K\left(r\right)}{r^{2}}$$

Comparing Equation (5) to Equation (2), we also have,

(6) $$\rho_{app}=P_{gap}\left(r\right)\frac{\Delta \beta }{A_{T}/\left(\pi r^{2}\right)}$$

Apparent reflectance, our range-independent and instrument-independent quantity, provides the objective variable for our lidar calibration for the canopy structure study for two reasons. First, $\rho_{app}$ can be modeled to derive canopy radiative and structural information. According to Equation (6), $\rho_{app}$ includes $P_{gap}\left(r\right)$, as determined by the structural characteristics of vegetative facets in the canopy, and Δβ, as determined by both the radiative and structural characteristics of the vegetative facets of the canopy. Second, $\rho_{app}$ can be interpreted as the reflectance value of a diffusely-reflecting, partly-absorbing panel filling the laser beam orthogonally that would return the same laser energy received by the lidar as the actual target at the same range. It can be realized as the ratio of (dark current corrected) ΔJ, the lidar intensity from a target to $\Delta J_{w}$, the intensity from a white Lambertian panel (orthogonal to the laser beam, reflectance $\rho_{w}=1$) at the same range, as shown in Equation (7):

(7) $$\rho_{app\;}=\frac{\Delta J}{\Delta J_{w}}\cdot \rho_{w}=\frac{\Delta J}{\Delta J_{w}}$$

Thus, $\rho_{app}$ is theoretically useful for the canopy structure study, as well as practically realizable for lidar calibration. It was introduced in Parkin *et al.* [58] and used in data interpretation by Jupp *et al.* [17].

#### 2.1.2. Physical Interpretation of Apparent Reflectance

When the thickness of the angular voxel Δr→0, we have the differential form of Equation (2) as:

(8) $$J\left(r\right)=\frac{\partial J}{\partial r}=J_{0}P_{gap}\left(r\right)\cdot \beta \left(r\right)\cdot K\left(r\right)\cdot \eta_{sys}\eta_{atm}\;\beta \left(r\right)=\frac{\partial \beta }{\partial r}=\int_{\Omega_{T}}\frac{1}{\pi }\Gamma \left(r,\Omega_{i}\to \Omega_{v}\right)d\Omega_{v}\cdot u_{L}\left(r\right)$$

where J(r) ($J\cdot m^{-1}$) is the received laser energy from range r per unit length of laser beam travel; β(r) ($m^{-1}$) is the effective volume backscatter ratio at range r, *i.e.*, the proportion of the incident laser radiation energy that is scattered back into the solid angle subtended by the telescope receiving area at range r per unit length of laser beam travel. The quantity $\left(1/\pi \right)\Gamma \left(r,\Omega_{i}\to \Omega_{v}\right)\cdot u_{L}\left(r\right)$ (${sr}^{-1}\cdot m^{-1}$) is the volume scattering phase function, which defines the part of the radiant energy onto a unit volume of vegetative facets in the direction $\Omega_{i}$ that is scattered in the direction $\Omega_{v}$ within the unit solid angle. The quantity β(r) is the integral of the volume scattering phase function over the solid angle subtended by the telescope aperture from range r. Accordingly, we have the differential apparent reflectance $\rho_{app}\left(r\right)$ ($m^{-1}$) from Equations (5) and (6), respectively.

(9) $$\rho_{app}\left(r\right)=J\left(r\right)\cdot \frac{r^{2}}{\Phi_{0}\cdot K\left(r\right)}$$

(10) $$\rho_{app}\left(r\right)=P_{gap}\left(r\right)\frac{\beta \left(r\right)}{A_{T}/\left(\pi r^{2}\right)\;}$$

Equation (9) shows how to practically calculate and interpret apparent reflectance from the received laser energy. Equation (10) shows how to theoretically model apparent reflectance from the radiative and structural characteristics of the canopy. To separate the radiative and structural information about canopy from $\rho_{app}\left(r\right)$, we assume Lambertian facets and the same diffuse reflectance $\rho_{d}$ for all vegetative elements that contribute to the received laser energy from which $\rho_{app}\left(r\right)$ is calculated. Then, $\left(1/\pi \right)\Gamma \left(r,\Omega_{i}\to \Omega_{v}\right)$ can be approximated by $G^{2}\cdot \rho_{d}/\pi$ (see Appendix A1 for the derivation). From Equations (8) and (9), we have,

(11) $$\rho_{app}\left(r\right)=P_{gap}\left(r\right)\cdot G^{2}\cdot u_{L}\left(r\right)\cdot \rho_{d}$$

Applying the differentiation of Equation (1) to the above,

(12) $$P_{hit}\left(r\right)=-\frac{\partial P_{gap}\left(r\right)}{\partial r}=P_{gap}\left(r\right)\cdot G\cdot u_{L}\left(r\right)$$

(13) $$\rho_{app}\left(r\right)=-\frac{\partial P_{gap}\left(r\right)}{\partial r}\cdot G\cdot \rho_{d}$$

Here, $P_{hit}\left(r\right)$ ($m^{-1}$) is the laser beam interception density [53], *i.e.*, the interception fraction by vegetative facets per unit length along the laser beam. Taking the integral over range on both sides of the Equation (13),

(14) $$\int_{0}^{r}\rho_{app}\left(r^{\prime }\right)dr^{\prime }=\left(1-P_{gap}\left(r\right)\right)\cdot G\cdot \rho_{d}$$

Thus, we can estimate $P_{gap}$ over range from the integral of differential apparent reflectance over range calculated with Equation (9) from received laser energy if the *G*-function and leaf diffuse reflectance are known. The gap probability with range is an important function for indirect measurement of canopy structure, such as LAI, clumping index and foliage profile [17,59,60]. Calibrating lidar return intensity to apparent reflectance enables better estimates of gap probability than just counting numbers of points returned from targets along a laser beam [17].

Note that Equation (14) omits the laser beam direction and implies two assumptions: (1) the *G*-function is constant over range; and (2) vegetative facets are all Lambertian with the same diffuse reflectance $\rho_{d}$. These two assumptions might not be true for vegetative elements traversed by a single laser beam. In practice, $\rho_{app}\left(r\right)$ from multiple laser shots are often averaged together (e.g., over all azimuth angles within zenith angle ranges), which will reduce the variance in estimating $P_{gap}\left(r\right)$ for the canopy as a whole.

The foregoing discussion has not considered wavelength (λ) in the lidar equation and $\rho_{app}$. From Equation (11), it is clear in the case of a bi- or multi-spectral lidar that $\rho_{d}\left(\lambda \right)$ of the scattering volume may be inferred from $\rho_{app}\left(\lambda \right)$, where the bi- or multi-spectral pulse encounters the same scattering volume at the same range and from the same direction. The difference in $\rho_{app}\left(\lambda \right)$ of a scattering volume is theoretically caused only by the spectral reflectance $\rho_{d}\left(\lambda \right)$, as the other terms in Equation (11) describe structural characteristics independent of wavelength. This conveniently provides a mechanism for the discrimination of different types of scattering materials based on their bi- or multi-spectral reflectance values $\rho_{d}\left(\lambda \right)$.

#### 2.1.3. Telescope Efficiency

The telescope efficiency function K(r) is needed by geometric laser systems using a telescope to focus the return power. It arises because the telescope is focused at infinity, and near-range objects are thus out of focus at the detector, which reduces the energy falling on the detector. The function is theoretically zero at zero range (the focal point of the telescope) and rises to unity at the range at which the focused return beam falls entirely within the detector. K(r) is usually omitted in the airborne lidar equation [39] because ground targets are sufficiently far enough from the instrument for K(r) to reach unity. For terrestrial lidar, many returns are from near-range targets, which requires including K(r) [30,50].

### 2.2. Recorded Return Waveforms and Apparent Reflectance

#### 2.2.1. Recording Return Waveforms

The basic lidar equation above describes return laser energy (J) by assuming an impulse of outgoing laser energy. For a pulsing laser, the actual recording of return power over time, *i.e.*, the return waveform, further involves: (1) the spatial distribution of outgoing laser energy (non-uniform laser beam cross-section, [61,62]); (2) the temporal shape of the outgoing laser pulse, $P_{0}\left(t\right)$ ($J\cdot s^{-1}$) (the spread of $J_{0}$ over a finite time); and (3) the characteristics of the detector-amplifier system of the instrument.

We shall not model the effects of the nonuniform beam cross-section on return waveforms here, because in processing terrestrial lidar data for the gap probability estimate, returns from many laser shots are usually aggregated together, which greatly reduces any variance due to the nonuniform shape of the beam cross-section.

We may express the pulse shape as a function of range, $P_{0}\left(r\right)$ ($J\cdot m^{-1}$), by converting the time to apparent range with $r=c\left(t-t_{p}\right)/2$, where $t_{p}$ is the time at which the outgoing pulse peak occurs and c is the speed of light. As $J_{0}$ in Equation (4) changes to $P_{0}\left(r\right)$, the term $\Phi_{0}$ becomes $\Phi_{0}\left(r\right)$, and the return signal P(r) becomes the convolution of $\rho_{app}\left(r\right)$ and $\Phi_{0}\left(r\right)$:

(15) $$P\left(r\right)=\int_{0}^{r}\Phi_{0}\left(r-r^{\prime }\right)\cdot \frac{\rho_{app\;}\left(r^{\prime }\right)\cdot K\left(r^{\prime }\right)}{{r^{\prime }}^{2}}dr^{\prime }=\Phi_{0}\left(r\right)*\frac{\rho_{app\;}\left(r\right)\cdot K\left(r\right)}{r^{2}}\;\Phi_{0}\left(r\right)=P_{0}\left(r\right)\frac{A_{T}}{\pi }\eta_{sys}\eta_{atm}$$

As P(r) is altered by the detector-amplifier system, the final recorded return waveform in digital counts I(r) is,

(16) $$I\left(r\right)=S_{R}\left(r\right)*\left[\frac{\rho_{app}\left(r\right)\cdot K\left(r\right)}{r^{2}}\right]$$

where $S_{R}\left(r\right)$ is the result of $\Phi_{0}\left(r\right)$ being altered by the detector-amplifier system [63].

#### 2.2.2. Modeling Return Waveforms

To get $\rho_{app}\left(r\right)$, we need to deconvolve $S_{R}\left(r\right)$ from I(r). However, deconvolution is very sensitive to noise. To reduce the effects of noise on data interpretation, we modeled $\rho_{app}\left(r\right)$ as a sequence of Dirac delta functions marking $N_{h}$ ($N_{h}\geq 1$) scattering clusters of vegetative facets corresponding to one or multiple return pulses in a waveform. This so-called delta-sequence model conceptually distributes vegetative facets in the j-th ($j=1\cdots N_{h}$) cluster at the range of $R_{j}$ inside a very thin angular voxel (*i.e.*, Δr→0); that is, ${\rho_{app}}_{j}\left(r\right)$ is a Dirac delta function, and a scattering cluster is a target with spatial extent of zero,

(17) $$\rho_{app_{j}}\left(r\right)={\rho_{app}}_{j}\left(R_{j}\right)\delta \left(r-R_{j}\right)$$

The apparent reflectance of the j-th voxel, $\rho_{app_{j}}$, which is the integral of differential apparent reflectance over range, is given by,

(18) $$\rho_{app_{j}}=\int^{}{\rho_{app}}_{j}\left(r\right)dr=\int^{}\rho_{app_{j}}\left(R_{j}\right)\delta \left(r-R_{j}\right)dr=\rho_{app_{j}}\left(R_{j}\right)$$

Using the delta-sequence model, I(r) is given by:

(19) $$I\left(r\right)=\sum_{j=1}^{N_{h}}\psi_{j}\left(r\right)\;\psi_{j}\left(r\right)=S_{R}\left(r-R_{j}\right)\cdot \frac{\rho_{app_{j}}\cdot K\left(R_{j}\right)}{R_{j}^{2}}$$

where $\psi_{j}\left(r\right)$ is the *j*-th return pulse resolvable from a return waveform. The peak intensity of $\psi_{j}\left(r\right)$, $\alpha_{j}$ is,

(20) $$\alpha_{j}=\psi_{j}\left(R_{j}\right)=S_{R}\left(0\right)\cdot \frac{\rho_{app_{j}}\cdot K\left(R_{j}\right)}{R_{j}^{2}}$$

For a given detector-amplifier system, $S_{R}\left(0\right)$ is a constant, denoted as $C_{0}$. Thus, the peak intensity of a return pulse, $\alpha_{j}$, is directly related to our calibration objective, apparent reflectance $\rho_{app_{j}}$ of an angular voxel filled with vegetative facets as follows,

(21) $${\rho_{app}}_{j}=\alpha_{j}\cdot \frac{R_{j}^{2}}{C_{0}\cdot K\left(R_{j}\right)}$$

If the spatial extent of a target is not zero (violation of the Dirac delta model), $\int^{}{\rho_{app}}_{j}\left(r\right)dr$ will be larger than ${\rho_{app}}_{j}\left(R_{j}\right)$. In other words, the actual apparent reflectance of the angular voxel will be larger than the value calculated from the Equation (21). This underestimate of ${\rho_{app}}_{j}$ due to the assumption of the Dirac delta model for clusters of vegetative facets can be compensated by correcting $\alpha_{j}$ according to the shape of return pulse $\psi_{j}\left(r\right)$ and will be pursued in future.

## 3. Instrument and Data Preprocessing

### 3.1. The Dual Wavelength Echidna Lidar

The scientific objective of DWEL instrument design is to separate leaves and woody materials in forests readily in three-dimensional space using their different spectral reflectance values at near-infrared (NIR, 1064 nm) and shortwave infrared (SWIR, 1548 nm) wavelengths. At the SWIR wavelength, the laser power returned from leaves is much lower than from woody materials, such as trunks and branches, due to absorption by liquid water in leaves. In contrast, returned power from leaves and woody materials is similar at the NIR wavelength. The two infrared lasers emit unpolarized pulses with a full-width half-maximum (FWHM) of 5 ± 0.1 ns; the two laser beams are aligned coaxially to less than 1 mrad. The collimated beam diameters of the two lasers are 6 mm. The beam divergences of both lasers are 2.5 mrad for the DWEL scans presented here. The laser scanning step is 2 mrad, slightly smaller than the beam divergence, which ensures continuous coverage of the hemispheres. A third continuous-wave green marker laser is also aligned with the two infrared signal lasers; since it is readily visible, it is used to position the triple beam or mark the scan path in the laboratory. More details of the DWEL instrument design and specifications are presented in [30,38].

#### 3.1.1. Internal Calibration Objects

Two scattering objects are fixed to the instrument to calibrate range and outgoing laser intensity. First, a fine stainless steel (removable) wire crosses the edge of the outgoing beam before it hits the scan mirror, thus scattering a small fraction of each outgoing pulse into the telescope and detectors. This allows a small part of the outgoing laser pulse to be present in the recorded signal, which assures the temporal alignment of individual waveforms and gives range precision values of one-sigma error of 4.75 cm at 1064 nm and of 2.33 cm at 1548 nm [38]. Second, a small circular Spectralon^®^ panel with nominal reflectance of 0.99 is affixed to the case so that each mirror rotation will acquire samples of outgoing pulses from this fixed target at a fixed range. These sampled waveforms are used primarily to track drifts in laser output power that occur through the scan, but can also establish the mean outgoing pulse times of the lasers for each mirror rotation in the absence of a wire signal or refine the temporal alignment of waveforms by a wire signal.

#### 3.1.2. Signal Recording and System Response

**Figure 1:**
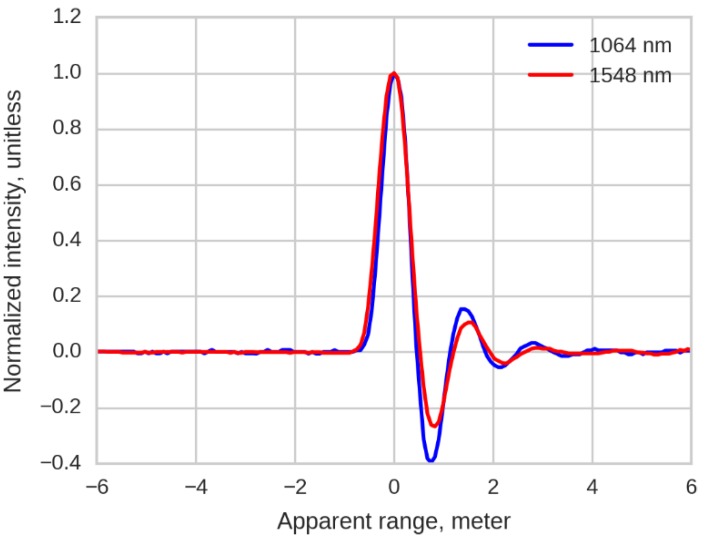
DWEL system response pulse. The two pulse peaks at the two wavelengths are aligned.

Figure 1 shows the mean of multiple samples of $S_{R}\left(r\right)$ given DWEL’s system response at each wavelength after background noise is removed. The pulses are normalized by peak intensity. The “ringing” response after the maximum is produced by the modulation transfer function of the combined detector-amplifier.

### 3.2. Preprocessing of DWEL Waveform Data

Before extraction of return pulse peaks and radiometric calibration, the raw waveforms from DWEL are preprocessed to: (1) remove background noise; (2) convert digitizer time to apparent range by aligning each waveform to the peak of the outgoing pulse using the signals from the scattering wire or internal Spectralon panel; (3) detect and correct saturated return pulses (see Section 3.2.1); (4) correct laser power drift, typically due to instrument temperature change, by scaling recorded intensities according to mean intensities observed from the internal Spectralon panel for each mirror rotation; and (5) calculate cross-covariance between waveforms and the system response function. This cross-covariance function changes the original asymmetric return pulses I(r) seen in each DWEL waveform to symmetric pulses. The new waveform of symmetric return pulses $I_{p}\left(r\right)$ is written as (⋆ denotes cross-correlation),

(22) $$I_{p}\left(r\right)=S_{R}\left(r\right)⋆I\left(r\right)=\varphi \left(r\right)*\left[\frac{\rho_{app}\left(r\right)\cdot K\left(r\right)}{r^{2}}\right]$$

where $\varphi \left(r\right)=S_{R}\left(r\right)⋆S_{R}\left(r\right)$ is a symmetric pulse after cross-covariance calculation. The preprocessed waveform $I_{p}\left(r\right)$ then provides the input to point cloud generation, thus avoiding pulse peak shifts due to the asymmetry of the original DWEL return pulse in later processing. An additional significant benefit of this operation is that it reduces uncorrelated noise and increases the signal-to-noise ratio prior to extraction of the signal in later processing.

#### Saturation Correction

Terrestrial laser scanners, in contrast to airborne scanners, will provide returns from close targets, sometimes within one meter for a placement in a forest with a dense or patchy understory, while also detecting targets at ranges of 100 m or more. This large relative variation in range provides a wide variation in return power that can exceed the limits of detector-amplifier systems (linear or nonlinear) and/or digitizers available for terrestrial scanners, and as a result, close targets can produce saturated pulse waveforms. Moreover, direct solar irradiance or specular reflectance may also produce saturated waveforms. The result may be either detector saturation, which produces an overloaded signal that persists through multiple digitizer bins or even multiple pulses, or digitizer saturation, which produces a flat-topped return pulse as the return signal exceeds the quantization range of the digitizer. In either case, the result is an unusual return pulse shape that cannot be used in calibration or to generate a scattering point with a correct apparent reflectance value.

In the DWEL instrument, detector saturation occurs in the rare case of a pulse striking an orthogonal specular target or corner reflector; normal target returns are well within the incoming power bounds of the DWEL’s detector-amplifier (Thorlabs PDA10CF) given the outgoing laser energy of DWEL. If the field of view of the telescope includes the Sun or the Sun’s aureole, the pulse may be lost completely as the detector and/or digitizer saturates or records high levels of continuous noise through the entire waveform. This situation is easy to detect, and such waveforms are identified as solar-saturated.

**Figure 2:**
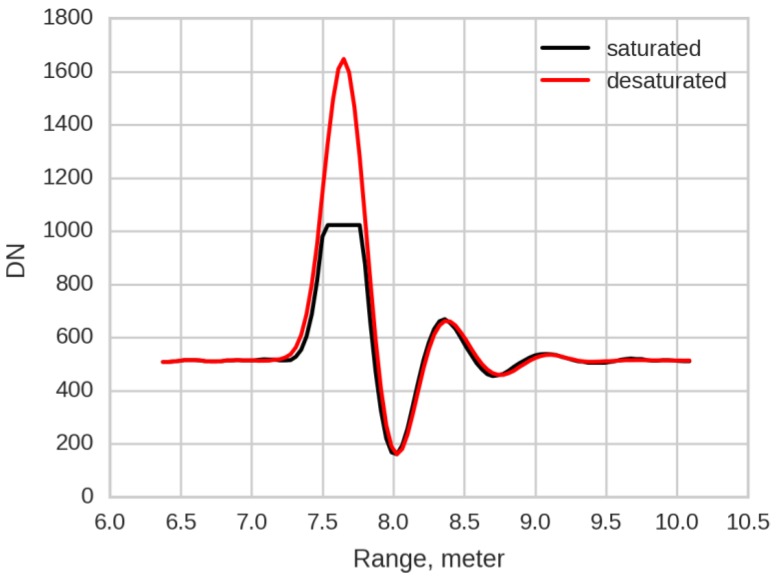
Example of saturated pulse and saturation correction.

Digitizer saturation, however, is commonly encountered in pulses returned from near objects. Here, a “saturation correction” is employed (Figure 2). Saturation creates a flat-topped pulse as the digitizer reaches its limit; however, the side-lobe trough and secondary peak are recorded correctly. By comparing saturated and unsaturated waveforms acquired from targets with high and low reflectance at the same range, we determined empirical ratios between the magnitudes of the side lobes and the unsaturated peak. These ratios are used to generate a pulse peak that is located at the mean range of the saturated bins. This pulse is identified as a “desaturated” pulse (saturation fixed) for further processing.

## 4. Radiometric Calibration Procedures

### 4.1. Calibration Model Setup

The calibration needs to mathematically model two important components of the lidar equation, the telescope efficiency function K(r) and the negative exponential fall-off of return intensity with range. To set up the calibration model, we may choose between two alternatives: a physical model based on the optical design of the instrument or an empirical model designed to best fit the data. A physical model describing the returned power of the DWEL instrument was derived from first principles [30].

However, initial tests of this model with the calibration data were not satisfactory. While the general shape of the response function fits the observations, the model showed significant departures from the behavior actually observed for the instrument, especially at near range. We believe that second-order effects, such as imperfect alignment interacting with the Gaussian beam cross-section, departure of divergence from nominal specifications or unmodeled electronic effects, were responsible for this variance.

Accordingly, we used a semi-empirical model to fit the data. According to Equation (21), for each extracted point with range R and digital count intensity α, we need the constant $C_{0}$ and the function K(r) to calculate apparent reflectance $\rho_{app}$. To find these unknowns, we require a collection of data points of $\left(R_{j},\;\alpha_{j},\rho_{app_{j}}\right)$ from targets of different reflectance values at multiple ranges. The quantities *R_j_* and $\alpha_{j}$ are derived from the extraction of pulse peaks; apparent reflectance $\rho_{app_{j}}$ may be taken as the diffuse reflectance $\rho_{d}$ of an extended Lambertian target held perpendicular to the laser beam. The telescope efficiency function K(r) (see Section 2.1.3) is modeled with a generalized logistic function [64]. The empirical calibration function for the DWEL is thus:

(23) $$\rho_{app}\left(R_{j}\right)=\alpha_{j}\cdot \frac{R_{j}^{b}}{C_{0}\cdot K\left(R_{j}\right)}\;K\left(r\right)=\frac{1}{{\left(1+C_{1}\cdot e^{-C_{2}\cdot r}\right)}^{C_{3}}}$$

where five parameters need to be estimated for the DWEL calibration, $\left(C_{0},C_{1},C_{2},C_{3},b\right)$. Of the three parameters for K(r), $C_{1}$ and $C_{3}$ together affect the range at which the function approaches its asymptote of one; $C_{2}$ controls the rate at which telescope efficiency rises from zero to one in the near range.

Note that the exponent of range is now taken as a variable, b, for two reasons. First, a calibration target surface, for example a manufactured Spectralon Lambertian panel, may not provide perfectly isotropic diffuse reflectance [65]. An anisotropic target may preferentially reflect radiation into a small solid angle in the direction of the instrument’s observation (*i.e.*, it may be specular, to some degree). Then, from Equation (2), a smaller $\Omega_{T}$ at farther ranges would yield an integral over directions where the area scattering function, $\left(1/\pi \right)\Gamma \left(r,\Omega_{i}\to \Omega_{v}\right)$, has higher values, thereby causing a larger Δβ and larger return energy J than if the target were perfectly isotropic. However, the reflectance value of a calibration panel is typically taken as a constant, for example measured by an integrating sphere, and is assumed to be isotropic in the calibration model. From Equation (5), if $\rho_{app}$ is kept constant, but J becomes larger, the exponent of range r will be smaller to compensate. Second, previous studies have also suggested that the exponent may need to accommodate electronic effects [48]. The exponent has therefore been treated as a variable in the calibration. Although the number of model parameters is large and they are not independent of each other, the calibration function can be fitted across its full range of application, thus avoiding issues of extrapolation beyond the limits of the fitting.

### 4.2. Calibration Data Collection

**Figure 3:**
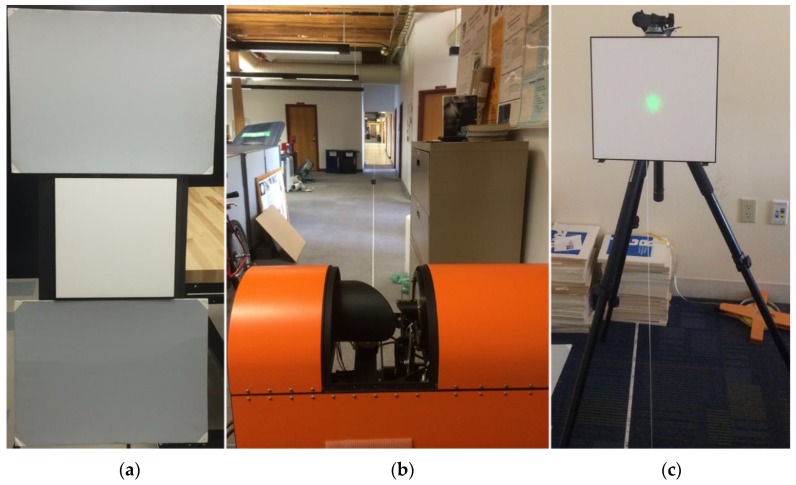
Calibration data collection. (**a**) Three panels for calibration, from top to bottom: painted light gray panel, white Spectralon panel and painted dark gray panel; (**b**) DWEL was set up in stationary mode with the laser pointing along the measuring tape laid out on the floor; panels were placed at 33 range locations along the tape; (**c**) the green laser was used to point the infrared lasers to the center of the panels.

To acquire the calibration data, three panels of different reflectance values (Figure 3a) were scanned by the DWEL from a nearly perpendicular direction at 33 range locations from 0.5 m to 70 m (Table 1). The range sampling intervals were based on a provisional calibration, made at the time of commissioning, that established the general shape of the K(r) curve. The instrument was set in stationary mode, *i.e.*, without the scan mirror or azimuth platform rotation (Figure 3b). The green marker laser was used to manually point the co-aligned lasers to the center of each panel at each placement (Figure 3c). The panel sizes are large enough to intercept the whole laser beam at 70 m. For each panel at each range, we collected around 150,000 waveform samples as candidates for calibration model fitting and evaluation.

The three panels included a white Spectralon panel and two foam boards painted with flat interior wall paint in light and dark gray tones derived by mixing black and white paints together. The gray panels provide unsaturated returns at near ranges. The $\rho_{app}$ value of the Spectralon panel uses the actual reflectance from the manufacturer’s specification. The $\rho_{app}$ values of the gray panels are calculated according to the Equation (7) as follows. First, we calculated the ratio of return intensities between gray panels (J) and the Spectralon panel ($J_{w}$) at each range, eliminating any saturated values. Then, we averaged the product values of the ratio and Spectralon panel reflectance at all ranges to obtain the $\rho_{app}$ values of the gray panels.

Table 2 shows the $\rho_{app}$ values and the measured reflectance by a FieldSpec Pro spectrometer (Analytical Spectral Devices) of the three panels. The difference between the $\rho_{app}$ values and the measured reflectance values for the gray panels is most likely caused by two effects. First, the spectrometer measures reflectance at 0° incidence angle and 10° view angle rather than by retroreflection, and the values appeared to be underestimated slightly due to reflectance anisotropy. Moreover, the reflectance of flat wall paint may have changed with time as the paint slowly cured between the spectrometer measurements and the acquisition of calibration data (about 20 weeks).

### 4.3. Calibration Model Fitting

To take advantage of the difference in spectral reflectance values of leaves and those of other targets at the NIR and SWIR bands, we not only try to minimize errors in $\rho_{app}$ at individual wavelengths in calibration model fitting, but also try to ensure the two wavelengths have the same or similar relative errors in $\rho_{app}$ in order to minimize artificial variations in spectral difference due to different errors in $\rho_{app}$ at the two wavelengths. Thus, we estimate the calibration parameters of NIR and SWIR bands together in a joint calibration model. The objective error function for the fitting of this model includes both relative errors in $\rho_{app}$ from individual wavelengths and spectral constraints from both wavelengths as follows:

(24) $$\begin{array}{ccc} f\left(\underset{˜}{C}\right) & = & f_{1}\left(\underset{˜}{C}\right)+f_{2}\left(\underset{˜}{C}\right)\\ f_{1}\left(\underset{˜}{C}\right) & = & \overset{N_{f}}{\underset{i=1}{\sum }}{\left(\frac{\hat{\rho_{Ni}}-\rho_{Ni}}{\rho_{Ni}}\right)}^{2}+\overset{N_{f}}{\underset{i=1}{\sum }}{\left(\frac{\hat{\rho_{Si}}-\rho_{Si}}{\rho_{Si}}\right)}^{2}\\ f_{2}\left(\underset{˜}{C}\right) & = & var\left(\hat{NDI}\right)+\overset{N_{f}}{\underset{i=1}{\sum }}{\left(\frac{\hat{\rho_{Ni}}+\hat{\rho_{Si}}-\rho_{Ni}-\rho_{Si}}{\rho_{i}+\rho_{Si}}\right)}^{2}\\ \hat{NDI} & = & \frac{\hat{\rho_{N}}-\hat{\rho_{S}}}{\hat{\rho_{N}}+\hat{\rho_{S}}} \end{array}$$

where $\underset{˜}{C}$ is the vector of five calibration parameters for NIR and five parameters for SWIR; $f\left(\underset{˜}{C}\right)$ is the objective error function as a sum of two components, including the error $f_{1}\left(\underset{˜}{C}\right)$ from individual wavelengths, and the spectral constraints $f_{2}\left(\underset{˜}{C}\right)$ from both wavelengths; subscript i represents the *i*-th data point, and subscript N and S represent NIR and SWIR; $N_{f}$ is the total number of data points used in calibration fitting; $\hat{\rho }$ is the apparent reflectance of panels estimated from the calibration model, while ρ is the actual apparent reflectance of panels; $\hat{NDI}$ is a normalized difference index to identify the spectral difference of target reflectance between NIR and SWIR; $var\left(\hat{NDI}\right)$ is the variance of NDI of data points in calibration fitting. In addition, we constrained the calibration parameters $C_{1}$ and $C_{3}$ to be equal across NIR and SWIR models. The objective error function $f\left(\underset{˜}{C}\right)$ has many local minima due to the high nonlinearity of the DWEL calibration model brought about by the K(r) function. We first used the genetic algorithm implemented in MATLAB [66] to search for initial parameter values that approach the global minimum. Then, we used the Nelder–Mead method [67] to reach the global minimum.

All of the return waveforms from the panels contain single returns at known nominal ranges. We searched maximum bins from the waveform sections around the nominal ranges and interpolated peaks using quadratic fitting of three bins around the maximum bin [68] to output intensities and ranges of these single returns. Saturated waveforms were excluded from calibration model fitting to avoid uncertainty from the saturation fix procedure. We randomly divided the remaining returns (about 24,000 samples for each range) into a training set (80 percent) and a validation set (20 percent). In the training set, return intensities were normalized by the corresponding panel reflectance to provide equivalent target reflectance values of 1.0 and then averaged together for each range to reduce noise in the data. Mean normalized intensities and mean ranges at 1064 nm and 1548 nm were paired according to panel range locations. Thirty pairs of data points from 1064 nm and 1548 nm were used to estimate the calibration parameters of 1064 nm and 1548 nm jointly by minimizing the error function $f\left(\underset{˜}{C}\right)$.

## 5. Results and Discussion

### 5.1. Radiometric Calibration

#### 5.1.1. Fitting of the Semi-Empirical Model

Table 3 provides values for the model coefficients derived from the model and procedure described in Section 4; they may be taken as examples, since recalibration will be necessary during the lifetime of the instrument.

**Figure 4:**
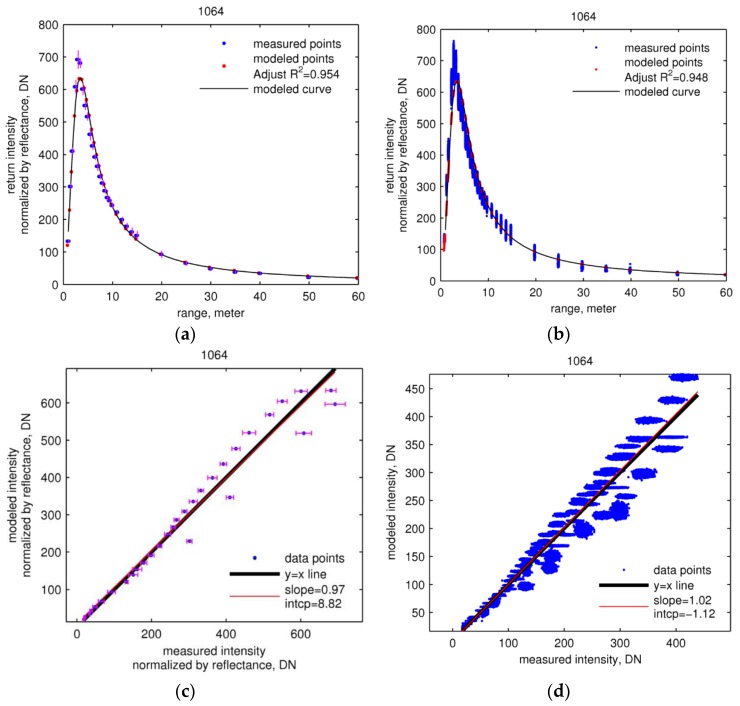
Estimation and validation of calibration of 1064 nm data. In both rows, the left column (**a,c**) shows the calibration function as fitted to training data, and the right column (**b,d**) shows the fit to the validation data. First row (a,b): measured and modeled intensity normalized by reflectance. Second row (c,d): scatter plots of measured against modeled intensity. The vertical error bars in (a) and horizontal error bars in (c) are one standard deviation of measured intensities normalized by reflectance.

**Figure 5:**
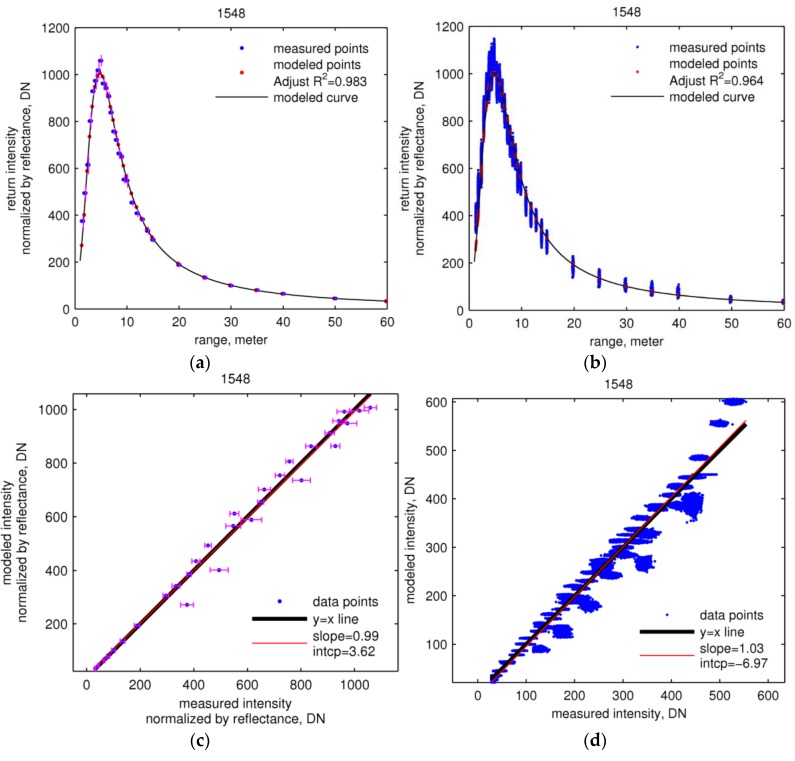
Estimation and validation of calibration of 1548 nm data. In both rows, the left column (**a,c**) shows the calibration function as fitted to training data, and the right column (**b,d**) shows the fit to the validation data. First row (a,b): measured and modeled intensity normalized by reflectance. Second row (c,d): scatter plots of measured against modeled intensity. The vertical error bars in (a) and horizontal error bars in (c) are one standard deviation of measured intensities normalized by reflectance.

Figure 4 and Figure 5 (first row) show the fits of the calibration functions (Equation (23)) for the two wavelengths to the training and validation data. The adjusted coefficient of determination ($R^{2}$) of modeled intensity at both wavelengths for both training and validation data (Table 4) indicates that the proposed calibration function and estimated parameters predict the return intensity well. The linear regressions between measured and modeled intensity for both training and validation data (Figure 4 and Figure 5, second row) yield values of slope very close to unity, as well as very small intercepts, indicating very good fits.

**Figure 6:**
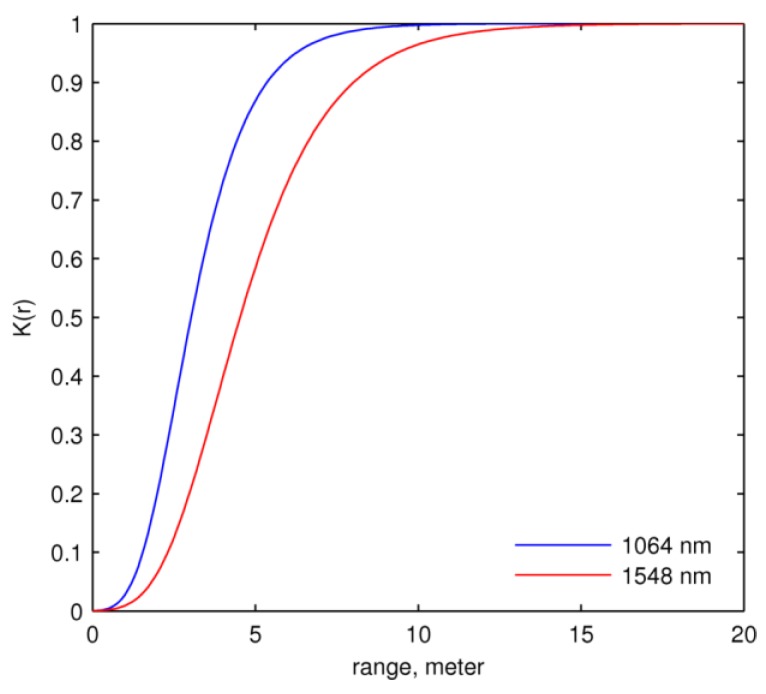
Telescope efficiency K(r) of the two wavelengths.

The calibration function curves in Figure 4 and Figure 5 (first row), which provide the return intensity of a target with unit reflectance, increase sharply and then fall exponentially. The normalized 1064 nm return intensity peaks at ~3.5 m (Figure 4, first row) and the 1548 nm peaks at ~5 m (Figure 5, first row). The curves of K(r), shown in Figure 6, rise from zero and plateau at about unity at ~10 m for the 1064 nm laser and ~15 m for 1548 nm.

#### 5.1.2. Apparent Reflectance Error and Its Sensitivity to Intensity and Range

**Figure 7:**
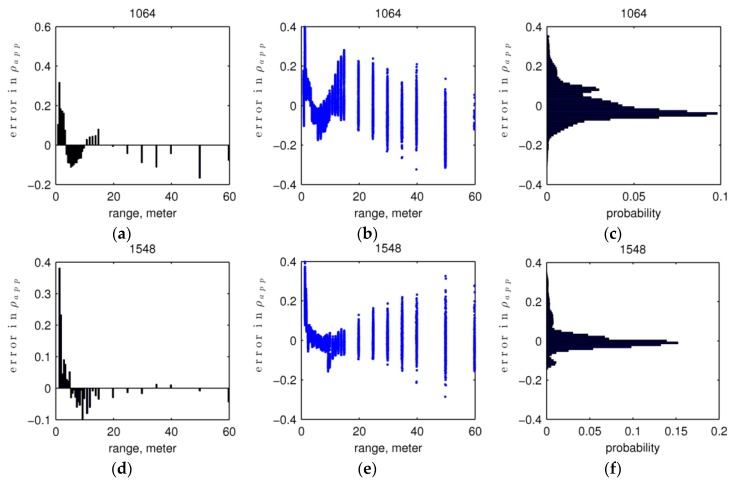
Errors in apparent reflectance. The first row (**a**–**c**) shows 1064 nm, and the second row (**d**–**f**) shows 1548 nm. The left column (a,d) is the deviation from calibration fitting with range. The middle (b,e) column is the deviation of validation points with range. The right column (c,f) is the histogram of deviations.

Because all intensities in calibration fitting and validation were normalized by corresponding panel reflectance values, the error in $\rho_{app}$ hereinafter means relative error, unless otherwise noted. The estimates of apparent reflectance $\rho_{app}$ by the calibration model from validation data show root mean squared errors (RMSE) of 0.092 at 1064 nm and 0.108 at 1548 nm (Table 4; Figure 7). The histograms of errors (Figure 7c,f) are centered near zero, which indicates little or no systematic offset in the apparent reflectance estimate.

The plots of errors in estimated $\rho_{app}$ against range for the validation dataset (Figure 7b,e) show larger and dispersed errors at very near range (<~3.5 m for 1064 nm and <~2 m for 1548 nm) and farther range (>~10 m for 1064 nm and >~20 m for 1548 nm), in contrast to smaller and less dispersed errors in between. This pattern of errors in $\rho_{app}$ over range is a combination of errors arising from range uncertainty (Δr) and return intensity uncertainty (Δα). We observed how Δr and Δα contribute to errors in $\rho_{app}$ separately with our calibration model over the range of our calibration data, 0.5 m to 70 m. We simulated $\delta \rho_{\alpha }$, the relative errors in $\rho_{app}$ only due to different return intensity uncertainty levels (±15 DN) at different ranges by keeping range error at zero. Then, we simulated $\delta \rho_{r}$, the relative errors in $\rho_{app}$ only due to different range uncertainty levels (±15 cm) at different ranges by keeping return intensity error at zero. These relative error ranges in the simulation of Δr and Δα are more than three-times larger than the standard deviation of range measurements and root mean squared noise in normalized intensities.

**Figure 8:**
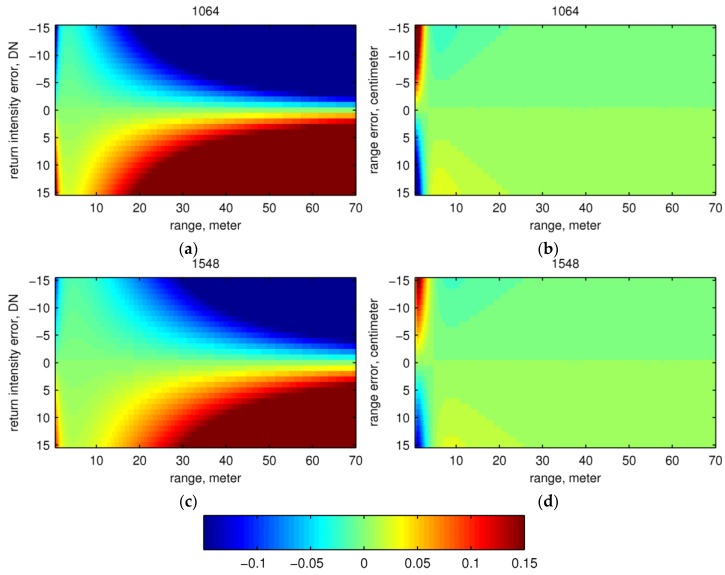
Sensitivity of the $\rho_{app}$ estimate on errors in return intensity and range. The image color shows relative error in the $\rho_{app}$ estimate (estimate − measurement). The color map scale is unified for all images for comparison purposes, but the actual error ranges of the four images are different and given here: (**a**) $\delta \rho_{\alpha }$ at 1064 nm [−0.928, 0.928]; (**b**) $\delta \rho_{r}$ at 1064 nm, [−0.226, 0.290]; (**c**) $\delta \rho_{\alpha }$ at 1548 nm, [−0.574, 0.574]; (**d**) $\delta \rho_{r}$ at 1548, [−0.133, 0.154].

Figure 8 presents a graphical display of the estimated relative errors calculated using the above procedure. The total relative error in $\rho_{app},\;\delta \rho$, can be approximated by $\delta \rho_{\alpha }+\delta \rho_{r}$ (see Appendix A2 for this derivation). Graphs (a) and (c) in Figure 8 show the relative error in apparent reflectance ($\delta \rho_{\alpha }$) produced by changes in return intensity (Δα) of ±15 digital counts (DN) (*y*-axis), as the error varies with range. At very near range (<~3 m), small changes in DN produce large errors in apparent reflectance; this effect arises because the telescope efficiency K(r) is very low and the return signal is weak. At near range between about 2 and 10 m, the signal is much stronger, and thus, the errors produced are fairly small (green colors). Between near and far range (10 to 70 m), the exponential decrease in signal provides a smooth transition from low sensitivity of apparent reflectance with DN error to high sensitivity. At far range (70 m), the signal is sufficiently diminished by fall-off with range that errors in apparent reflectance are large given a deviation of just a few counts from true values.

In contrast, large relative error in apparent reflectance ($\delta \rho_{r}$) produced by changes in range (Δr) of ±15 cm (Figure 8b,d) (*y*-axis) is limited to the very near range (<~5 m). The weak signal in this range provides large errors, which decrease rapidly as the telescope efficiency function K(r) increases the signal strength. Beyond this range, the relative error in apparent reflectance remains low.

From this analysis, we see that the error in apparent reflectance due to error in range ($\delta \rho_{r})$ dominates at near ranges, while the error due to return intensity ($\delta \rho_{\alpha }$) dominates at far ranges. The exact range at which $\delta \rho_{\alpha }$ surpasses δρ and becomes dominant depends on the uncertainty level of return intensity given the calibration model. Thus, we see larger and more dispersed errors at very near ranges in validation data (Figure 7b,e), mainly due to range uncertainty, and at far ranges, mainly due to return intensity uncertainty. The range accuracy is therefore more critical in the near-range target calibration, while the return intensity accuracy becomes more critical in the far-range target calibration.

The problem for calibration here is that returns from far ranges have a lower signal-to-noise ratio, but their calibration is highly sensitive to return intensity uncertainty. Thus, the noise level of lidar return intensity needs to be characterized to find the range at which the reflectance uncertainty δρ exceeds a desirable level given the calibration model. For returns from far ranges, the apparent reflectance should be used carefully. For returns from near ranges, δρ could be very high if the range uncertainty is not low enough. However, lidar instruments generally give range measurements of high accuracy.

### 5.2. Calibration Comparison of the Two Wavelengths

The two telescope efficiency functions K(r) at the two wavelengths (Figure 6) suggest different optical characteristics of the two beam pathways through the DWEL instrument. As noted earlier, each pathway uses a separate wavelength-dependent divergence optic and focusing lens in the detector assembly, which can produce small differences in beam width and detector field of view. Moreover, the two laser beams may not be exactly coincident due to small errors in alignment. As a result, the two functions show different shapes.

In addition, the range exponent values b for the two wavelengths are different, but both are smaller than the theoretical value of two that applies to scattering from a perfectly diffuse surface. As noted in Section 4.1, the observed value may depart from two for a number of reasons, including slight misalignment, electronic effects in the detector-amplifier-digitizer systems and the partial specularity of target surfaces. Moreover, as a free parameter in the model inversion, the range exponent may be adjusted by the nonlinear fitting procedure to better shape the telescope efficiency function. While a more physical model grounded in instrument optics and first principles of scattering might be desirable, an empirical model will capture the data trend more accurately in the face of physical and electronic unknowns. As shown above, our calibration functions fit the data well, predicting observed intensities from calibration targets and retrieving reflectance from observed intensities with low errors.

## 6. Conclusions

We present a thorough derivation of the calibration objective variable, apparent reflectance, from the basic scattering lidar equation using Ross’s framework of radiation regime modeling of the vegetation canopy. As an instrument-free measure, apparent reflectance facilitates merging point data from multiple instruments, allowing assessment of the effects of angular resolution and beam divergence on structure retrieval and even providing spectral information for scanners using different laser wavelengths.

Calibration of our full-waveform, dual-wavelength, terrestrial laser scanner presents a number of challenges relevant to the next generation of terrestrial laser scanners. The need to scan from near to far range requires characterizing both telescopic effects, which reduce the near-range signal with increasing proximity due to defocusing, and saturation effects, which alter the return pulse shape of near-range scattering events. We show how to overcome these challenges by formulating a flexible calibration model, acquiring appropriate calibration data, fitting the model with a constraint providing spectral consistency and testing the results and the sensitivity of errors to uncertainties in range and intensity. We also provide solutions to the problems of saturated returns, slow change in laser output pulse energy and variance in the timing of laser pulse emissions.

In addition, dual- or multi-wavelength data must be consistent in spectral performance. By using a semi-empirical calibration model fitted to data, it is not difficult to add a constraint that optimizes spectral “flatness” with range using a Lambertian target. This step is particularly useful for the DWEL, since the laser wavelengths are chosen specifically for their ability to separate hits of water-bearing leaves from hits of the dry bark of trunks and branches and dry ground surfaces.

The RMSE values (relative errors) of apparent reflectance from our calibration procedure, 8.1 percent for 1064 nm and 6.4 percent for 1548 nm, show that the parameterized model accurately converts lidar return intensities in digital counts to apparent reflectance. This calibration model can apply to almost any terrestrial lidar instrument using a telescope to focus the return power. A sensitivity analysis shows that the apparent reflectance error from radiometric calibration is dominated by range errors at near ranges, but by return intensity errors at far ranges.

While the calibration of a terrestrial lidar is by nature more difficult than that of an airborne lidar, one advantage is that controlled laboratory or field calibration measurements can be readily designed and executed. If stationary operation can fix the beam on the target panel, it is easy to acquire pulses at measured ranges in a long corridor or outdoor environment. If stationary operation is not possible, only short scan segments crossing the target need to be acquired. Calibration is also aided by having targets of different reflectance, so that darker panels can provide unsaturated signals at ranges of the greatest returns (e.g., 10 to 12 m for DWEL). While our painted panels functioned well, a set of Lambertian panels with well-characterized diffuse reflectance properties ranging from light to dark would be desirable.

The next step is to use calibrated data to retrieve forest structural parameters with the new dual-wavelength data, following the pathways pioneered with the heritage Echidna Validation Instrument, but extending them to new information from the Dual-Wavelength Echidna lidar. These are subjects of papers now in preparation.

## Figures and Tables

**Table 1:** Range sample design.

Range (m)	Range Interval (m)	Measurement Positions
[0.5, 10] *	0.5	20
(10, 15]	1	5
(15, 40]	5	5
(40, 70]	10	3

* “[” or “]” means inclusive of range boundary while “(” means exclusive of range boundary.

**Table 2:** Reflectance values of panels used in calibration.

Target	NIR Reflectance		SWIR Reflectance		Dimension (cm by cm)
	Measured ^1^	$\rho_{app}\;$ ^2^	Measured ^1^	$\rho_{app}\;$ ^2^	
White Spectralon panel ^3^	0.99		0.98		30.5 × 30.5
Gray Painted Panel 1	0.436	0.574	0.349	0.447	38.0 × 30.5
Gray Painted Panel 2	0.320	0.431	0.245	0.329	38.0 × 30.5

^1^ From the spectrometer with the illuminated probe; ^2^ calculated according to Equation (7); ^3^ manufacturer’s calibrated value.

**Table 3:** An example set of DWEL calibration parameters.

Parameter	Wavelength	
	1064 nm	1548 nm
C0	5788.265818	22,054.218342
C1	0.000319	0.000319
C2	0.808880	0.540762
C3	25,176.835032	25,176.835032
b	1.384297	1.585985

**Table 4:** Assessment of calibration fitting and validation.

Wavelength		1064 nm	1548 nm
Measured *vs.* Modeled Intensity, Adjusted $R^{2}$	Training ^1^	0.954	0.983
	Validation ^2^	0.948	0.964
RMSE of Apparent Reflectance	Training ^1^	0.108	0.092
	Validation ^2^	0.081	0.064

^1^ Thirty data points used for training by averaging unsaturated waveforms from three panels at 30 ranges (19,200 waveforms at each range); no good waveforms from three ranges (0.5, 1.0 and 70 m) out of 33 measured locations; ^2^ about 4800 waveforms used for validation. See Section 4.2 and Section 4.3 for more details.

## Appendix A

### A1. Area Scattering Phase Function for Lambertian Facets of the Same Diffuse Reflectance

Let $g_{L}\left(r,\Omega_{L}\right)$ (${sr}^{-1}$) be the probability function of leaf angle distribution at a location designated by range r along a laser beam of interest; the Ross *G*-function is [56],

(A1) $$G\left(r,\Omega \right)=\frac{1}{2\pi }\int_{\Omega_{1}}g_{L}\left(r,\Omega_{L}\right)\left|\cos\langle \Omega ,\Omega_{L}\rangle \right|d\Omega_{L}$$

where the integral interval $\Omega_{1}$ defines all of the possible leaf normal directions within an elemental volume at range r. Let $\left(1/\pi \right)\gamma_{L}\left(\Omega_{L},\Omega_{i}\to \Omega_{v}\right)$ be the leaf scattering phase function that defines the part of irradiance in the direction $\Omega_{i}$, which is scattered from the leaf unit area perpendicular to the direction $\Omega_{L}$ to the unit solid angle around the direction $\Omega_{v}$ [56]. The area scattering phase function is derived as,

(A2) $$\begin{array}{c} \frac{1}{\pi }\Gamma \left(r,\Omega_{i}\to \Omega_{v}\right)\\ =\int_{\Omega_{1}}\frac{g_{L}\left(r,\Omega_{L}\right)}{2\pi }\frac{\gamma_{L}\left(\Omega_{L},\Omega_{i}\to \Omega_{v}\right)}{\pi }\left|\cos\langle \Omega_{i},\Omega_{L}\rangle \right|\left|\cos\langle \Omega_{v},\Omega_{L}\rangle \right|d\Omega_{L} \end{array}$$

If all vegetative facets are Lambertian and have the same diffuse reflectance $\rho_{d}$, since lidar instruments make observations in the backscattering direction, *i.e.*, $\Omega_{v}=\Omega_{i}-\pi =\Omega$, then we have,

(A3) $$\frac{1}{\pi }\Gamma \left(r,\Omega_{i}\to \Omega_{v}\right)=\frac{\rho_{d}}{\pi }\int_{\Omega_{1}}\frac{g_{L}\left(r,\Omega_{L}\right)}{2\pi }{\left|\cos\langle \Omega ,\Omega_{L}\rangle \right|}^{2}d\Omega_{L}\approx \frac{\rho_{d}}{\pi }G{\left(r,\Omega \right)}^{2}$$

The two *G*-functions in the area scattering phase function correspond to the two cosine terms in the integral. One cosine term ($\left|\cos\langle \Omega_{i},\Omega_{L}\rangle \right|$) accounts for the projected area that intercepts incoming irradiance per unit vegetative facet area. The other cosine term ($\left|\cos\langle \Omega_{v},\Omega_{L}\rangle \right|$) accounts for the decrease of radiant intensity ($w\cdot {sr}^{-1}$) with the cosine, *i.e.*, projected area for a Lambertian surface. Furthermore, we assume the leaf angle distribution is constant within the canopy and so is the *G*-function and area scattering function.

(A4) $$\frac{1}{\pi }\Gamma \left(\Omega_{i}\to \Omega_{v}\right)\approx \frac{\rho_{d}}{\pi }G{\left(\Omega \right)}^{2}$$

### A2. Error in Apparent Reflectance from Two Sources: Range and Return Intensity

According to Equation (23), at a given range r,

(A5) $$\alpha =\frac{C_{0}K\left(r\right)}{r^{b}}\cdot \rho_{app}\;\rho_{app}=\frac{r^{b}}{C_{0}K\left(r\right)}\cdot \alpha$$

We omit the subscript j here for clarity. If the absolute errors in range r and return intensity α are Δr and Δα, respectively:

(A6) $$\Delta \rho_{r}=\frac{{\left(r+\Delta r\right)}^{b}}{C_{0}K\left(r+\Delta r\right)}\cdot \alpha -\rho_{app}$$

(A7) $$\Delta \rho_{\alpha }=\frac{r^{b}}{C_{0}K\left(r\right)}\cdot \left(\alpha +\Delta \alpha \right)-\rho_{app}=\frac{r^{b}}{C_{0}K\left(r\right)}\cdot \Delta \alpha =\frac{\rho_{app}}{\alpha }\cdot \Delta \alpha$$

where $\Delta \rho_{r}$ and $\Delta \rho_{\alpha }$ are errors in the apparent reflectance estimate due to Δr and Δα separately. Notice that $\Delta \rho_{r}$ and $\Delta \rho_{\alpha }$ both change with range. Now, the total absolute error in apparent reflectance, Δρ due to $\Delta \rho_{r}$ and $\Delta \rho_{\alpha }$, together is:

(A8) $$\Delta \rho =\frac{{\left(r+\Delta r\right)}^{b}}{C_{0}K\left(r+\Delta r\right)}\cdot \left(\alpha +\Delta \alpha \right)-\rho_{app}$$

Furthermore, according to Equations (A6) and (A7), we have:

(A9) $$\frac{{\left(r+\Delta r\right)}^{b}}{C_{0}K\left(r+\Delta r\right)}=\frac{\Delta \rho_{r}+\rho_{app}}{\alpha }\;\Delta \alpha =\alpha \cdot \frac{\Delta \rho_{\alpha }}{\rho_{app}}$$

Combining Equation (A8) and (A9),

(A10) $$\Delta \rho =\frac{\Delta \rho_{r}+\rho_{app}}{\alpha }\cdot \left(\alpha +\Delta \rho_{\alpha }\cdot \frac{\alpha }{\rho_{app}}\right)-\rho_{app}=\Delta \rho_{r}+\Delta \rho_{\alpha }+\frac{\Delta \rho_{r}\cdot \Delta \rho_{\alpha }}{\rho_{app}}$$

The relative errors in estimated apparent reflectance due to Δr and Δα separately are denoted as $\delta \rho_{r}=\Delta \rho_{r}/\rho_{app}$ and $\delta \rho_{\alpha }=\Delta \rho_{\alpha }/\rho_{app}$. Then, the total relative error in the apparent reflectance estimate due to Δr and Δα together is,

(A11) $$\begin{array}{ccc} \delta \rho & = & \frac{\Delta \rho }{\rho_{app}}\\ & = & \frac{1}{\rho_{app}}\left(\delta \rho_{r}\cdot \rho_{app}+\delta \rho_{\alpha }\cdot \rho_{app}+\frac{\delta \rho_{r}\cdot \rho_{app}\cdot \delta \rho_{\alpha }\cdot \rho_{app}}{\rho_{app}}\right)\\ & = & \delta \rho_{r}+\delta \rho_{\alpha }+\delta \rho_{r}\cdot \delta \rho_{\alpha } \end{array}$$

According to Figure 8, at near range, $\delta \rho_{r}$ is large, while $\delta \rho_{\alpha }$ is very small. At far range, it is the opposite. Thus, $\delta \rho_{r}\cdot \delta \rho_{\alpha }$ is small for any given range, and we can have,

(A12) $$\delta \rho \approx \delta \rho_{r}+\delta \rho_{\alpha }$$
